# Protein-protein interaction (PPI) network analysis reveals important hub proteins and sub-network modules for root development in rice (*Oryza sativa*)

**DOI:** 10.1186/s43141-023-00515-8

**Published:** 2023-05-29

**Authors:** Samadhi S. Wimalagunasekara, Janith W. J. K. Weeraman, Shamala Tirimanne, Pasan C. Fernando

**Affiliations:** Department of Plant Sciences, Faculty of Science, University of Colombo, Colombo, Sri Lanka

**Keywords:** Root development, Network analysis, Protein-protein interactions, Hub proteins, Sub-modules, Rice (*Oryza sativa*)

## Abstract

**Background:**

The root system is vital to plant growth and survival. Therefore, genetic improvement of the root system is beneficial for developing stress-tolerant and improved plant varieties. This requires the identification of proteins that significantly contribute to root development. Analyzing protein-protein interaction (PPI) networks is vastly beneficial in studying developmental phenotypes, such as root development, because a phenotype is an outcome of several interacting proteins. PPI networks can be analyzed to identify modules and get a global understanding of important proteins governing the phenotypes. PPI network analysis for root development in rice has not been performed before and has the potential to yield new findings to improve stress tolerance.

**Results:**

Here, the network module for root development was extracted from the global *Oryza sativa* PPI network retrieved from the STRING database. Novel protein candidates were predicted, and hub proteins and sub-modules were identified from the extracted module. The validation of the predictions yielded 75 novel candidate proteins, 6 sub-modules, 20 intramodular hubs, and 2 intermodular hubs.

**Conclusions:**

These results show how the PPI network module is organized for root development and can be used for future wet-lab studies for producing improved rice varieties.

**Supplementary Information:**

The online version contains supplementary material available at 10.1186/s43141-023-00515-8.

## Background

*Oryza sativa* has a very high demand as a staple food, and although much successful research has been carried out to improve the yield, it tends to decrease drastically in response to environmental stresses. Therefore, the improvement of *O. sativa* must continue, aiming at sufficient supply for the increasing population, which requires producing improved *O. sativa* varieties with higher yields and a higher ability to withstand environmental stresses [1–3].

The root system is the main component that supplies water and nutrients to the plant and plays a major role in withstanding environmental stresses [4]. Therefore, it should have a high priority when improving plant varieties. Root development is a complex biological process regulated by a collection of biological pathways, which are influenced by environmental and genetic factors [4]. This research is focused on investigating the genetic factors by identifying the functionally important proteins and their interactions responsible for root development in *O. sativa* using network analysis.

Biological processes are regulated by a collection of proteins and their interactions. These protein-protein interactions (PPI) are represented as PPI networks [5, 6]. PPI data are generated using wet-lab and computational techniques and are stored in databases [7, 8], such as DIP, STRING, and BioGRID. Among these databases, the STRING database is popular because of the higher abundance, coverage, and better quality control of PPI data [7, 9, 10]. STRING contains PPIs from both experimental computational methods and provides a combined quality score for each interaction by integrating the data from various resources such as literature and gene expression profiles [7, 10, 11]. Using this approach produces more accurate predictions compared to networks that solely rely on experimentally determined interactions, which are prone to high rates of false positives and false negatives in identifying interactions [10, 12]. PPI networks contain modules, which are distinct collections of proteins usually specific for a particular function or a phenotype [5, 13]. Hence, PPI networks can be analyzed to identify modules, which represent underlying protein interactions that determine the molecular functions and phenotypes. Furthermore, PPI networks are used for predicting novel protein candidates for molecular functions and phenotypes based on their interactions with known neighbors [14, 15]. Though wet-lab methods are available for predicting new protein candidates, computational methods for protein function prediction are faster, more cost-effective, and less laborious than wet-lab methods [16, 17]. Sequence similarity-based methods are popular computational approaches, which have been proven to be effective for some protein molecular function prediction studies, but they are less efficient for phenotype studies [16, 18]. This is because proteins associated with one phenotype can include proteins with highly diverse sequences, annotated with different molecular functions [12, 15, 18]. Therefore, predicting proteins for phenotypes using PPI networks is more accurate and comprehensive than sequence similarity-based methods [16, 18].

PPI network analysis can be used to identify the sub-modules within a particular module of a phenotype, and analysis of these sub-modules allows one to identify the related biological pathways and important proteins, i.e., hub proteins, involved in those pathways [19, 20]. Identifying the hub proteins of a module is an important advantage of performing network module analysis [15]. Hubs have a higher number of interactions compared to non-hubs [21]. There are two types of hubs: intramodular and intermodular hubs [22]. Intramodular hubs can be found with their partners within the functional modules, while intermodular hubs act between the modules and interconnect them [21–23]. Removal of a hub has a higher impact compared to non-hubs because it impacts several biological pathways in the network [22], which disrupts the resulting phenotype. Therefore, hub proteins are identified as important proteins that play a critical role in maintaining module organization and stability [24]. These are usually important drug targets in human-related studies [25] or genetic engineering targets in crop improvement [26].

PPI networks allow the understanding of the global organization of PPIs, sub-modules, connectivity among those sub-modules, and the hub proteins [8, 27, 28]. The interpretation of these networks reveals the biological pathways associated with a particular phenotype. The efficiency of PPI network analysis has been proven in human disease research [8, 29, 30], but to our knowledge, this method has never been used on root development in *O. sativa*.

This study aimed to apply PPI network analysis techniques used in other biological fields to analyze proteins involved in root development in *O. sativa*. This involved predicting potential novel candidates for root development, visualizing and identifying sub-modules, and analyzing their biological pathways. Additionally, this study also aimed to identify potentially important hub proteins related to root development and characterize the key interactions that are related to this function. The results shed light on how protein-protein interactions (PPIs) play a crucial role in root development, which can be useful for future studies aimed at improving root architecture and developing more stress-resistant varieties of *O. sativa*.

## Methods

### Data retrieval and preprocessing

The proteins already known to be involved in root development (seed proteins) were retrieved from the literature and the STRING database (version 11.0; July 2019; https://string-db.org/). The PPI network and supplementary data for *O. sativa* were downloaded from the STRING database (retrieved and downloaded on July 17, 2019).

To improve the reliability and the quality of the downloaded PPI network, it was filtered using the “combined score” according to a recommended cutoff mark of 400 [10]. Duplicates of the same record were removed, and STRING identifiers (IDs) for proteins were converted to preferred protein names to facilitate further analysis.

### Network-based candidate gene prediction and root development protein module extraction

The Hishigaki method was selected for the candidate gene prediction [14], and the prediction score was calculated according to the equation below.

$$prediction\, score= \frac{{\left({n}_{f\left(u\right)}- {e}_{f}\right)}^{2}}{{e}_{f}}$$

Here, *f* denotes the function of interest, and *u* denotes the protein of interest. The number of proteins with the function (*f*) in the n-neighborhood of *u* is given by *n*_f(u)_, and *e*_f_ denotes the expected frequency for the function calculated as follows:

$$ef=\frac{{tot}_{f} \times n(u)}{{tot}_{n}}$$

The total number of proteins annotated to the function of interest (*f*) in the network is denoted by *tot*_f_, and *tot*_n_ denotes the total number of proteins in the network; *n(u)* denotes the total number of proteins in the immediate neighborhood of the interested protein (*u*) [14].

Predicted scores were sorted to obtain the proteins with the highest predicted scores. The top 20, 50, 75, and 100 proteins were listed with seed proteins, and PPI modules for those lists were extracted from the preprocessed network and visualized using the Cytoscape software (version 3.7.1) [31, 32]. The final cutoff used for the rest of the analysis was chosen to maximize the number of seed proteins that were captured by the algorithm as well as to minimize the number of possible false positives that may result from less stringent cutoffs.

### Validation of the predictions

Computational validation of the predicted proteins was required to confirm the accuracy of the predictions. Validation was done using enrichment analysis and performing a literature search on the predicted proteins.

Enrichment analyses were performed using the DAVID (DAVID Bioinformatics Resources 6.8; https://david.ncifcrf.gov/home.jsp) web application. The functional annotation tool in DAVID was used, and the official gene symbol was selected as the identifier [33]. The biological process component of the Gene Ontology (GO-BP) and KEGG pathways, which had significant *p*-values (< 0.05), was selected for further analysis [33]. Literature searches were also used to further validate the predictions and the enriched biological pathways.

### Identification and analysis of sub-modules

Preliminary identification of sub-modules was done using MCODE (version 1.5.1) [34, 35] plug-in in Cytoscape software with the clustering parameters as follows: degree cutoff = 2, node score cutoff = 0.6, k-core = 2, and max. depth = 100, and further cluster expansions were done by observing the network module visualization.

Enrichment analysis and functional interpretation of sub-modules and hub proteins were performed using the DAVID enrichment analysis tool (DAVID Bioinformatics Resources 6.8) and literature mining.

### Identification and analysis of hub proteins

Intramodular hub proteins were selected according to the degree of each protein. The degree cutoff for hub selection was determined by analyzing the degree distribution and picking the top 10% of proteins with the highest degrees [21]. Furthermore, intermodular hubs were identified by analyzing the inter-modular connections which connect different sub-modules. Specifically, the proteins which connect at least 3 sub-modules were selected as intermodular hubs. Functional interpretations of hub proteins were performed by investigating the literature.

**Fig. 1:**
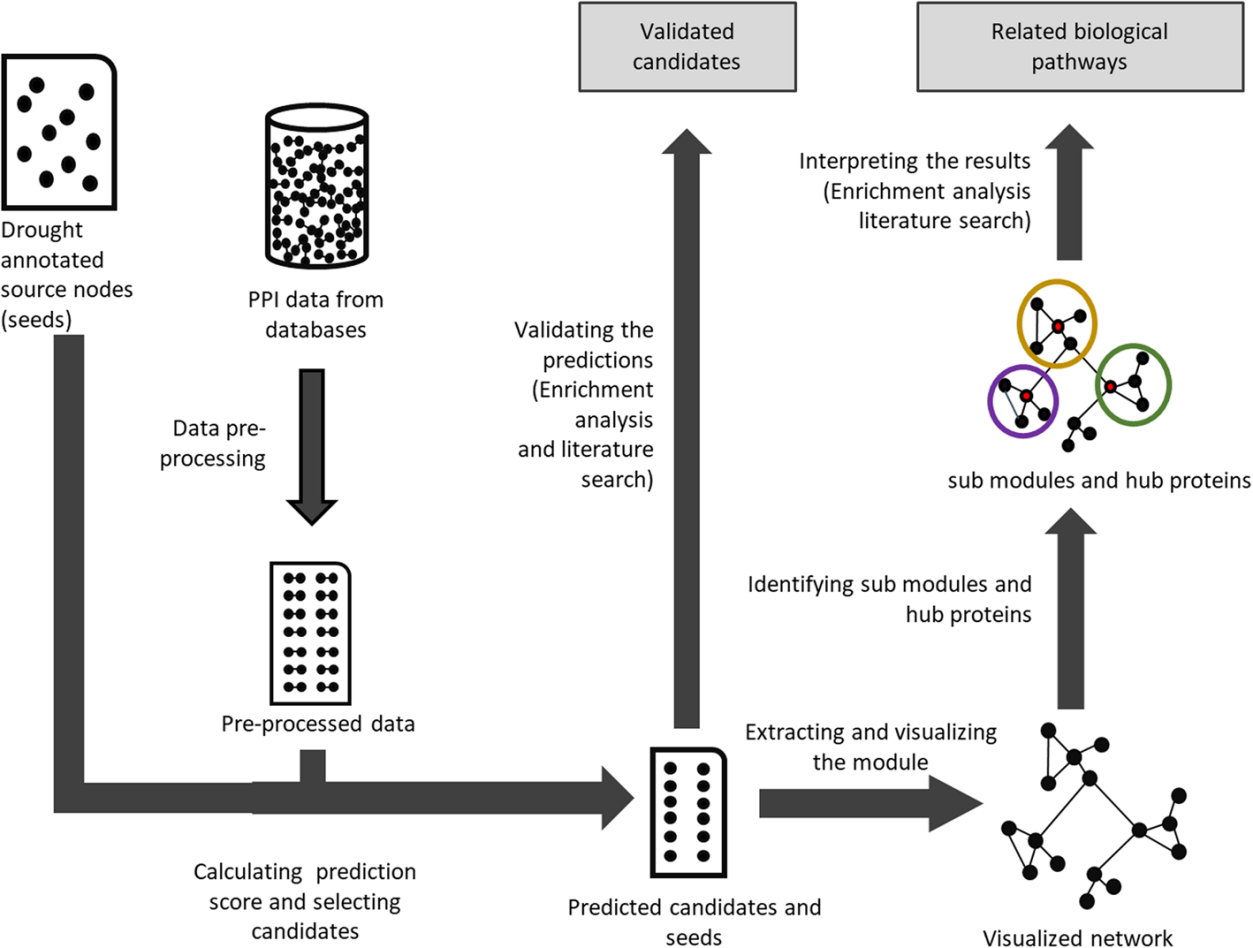
PPI network-based candidate protein prediction and validation workflow

The methodology of this study is briefly illustrated in Fig. 1.

All required scripts were written in Python (version 2.7) and deposited in a GitHub repository (https://github.com/Samadhi9/PPIN-analysis).

## Results

### Data retrieval and data preprocessing

Altogether, there were 51 seed proteins extracted from the literature and the STRING database (Supplementary Table S1). The *O. sativa* STRING PPI network contained 25,106 proteins and 8,949,048 interactions. There were 21,212 proteins and 1,608,106 interactions after filtering by > 400 combined score cutoff. The number of interactions was reduced to 803,817 after removing duplicates.

### Network-based candidate protein prediction and root development network module extraction

The Hishigaki method was used for network-based candidate protein prediction, and after several trials and errors, the top 75 candidates were selected as the most suitable number of candidates for further analyses because it gave the best visualization of the root development network module by connecting most of the sub-modules. Moreover, a significant number of seed proteins were included in the extracted network module (Table 1).

**Table 1:** The number of seed proteins that were present and absent in the root development network modules according to different thresholds of selecting protein candidates

	Top 20 candidates		Top 50 candidates		Top 75 candidates		Top 100 candidates	
	**Present**	**Absent**	**Present**	**Absent**	**Present**	**Absent**	**Present**	**Absent**
No. of seeds	38	13	41	10	45	6	45	6

**Fig. 2:**
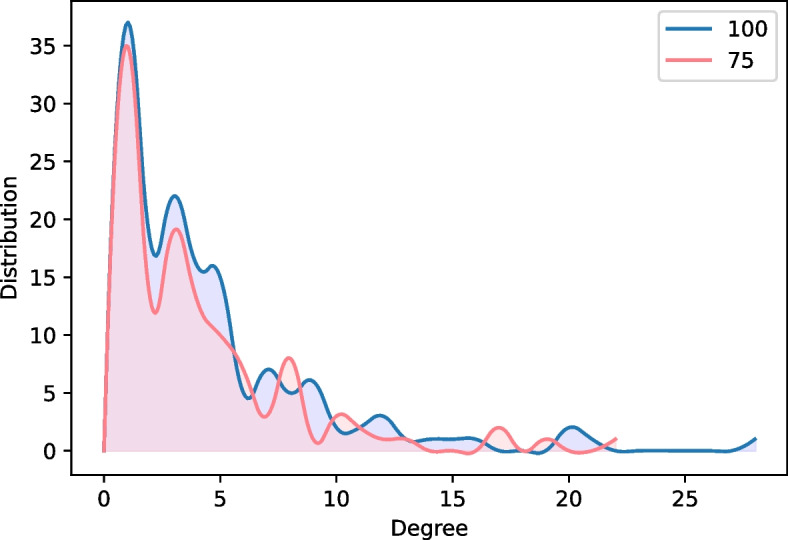
Degree distribution comparison between modules containing top 75 and 100 predicted proteins

As shown in Table 1, the top 20 and the top 50 candidates had a lower number of seeds present compared to the top 75 and 100. The top 75 and 100 had better seed retention, and both retained the same number (45) of seed proteins. Furthermore, degree distribution plots for the modules with 75 and 100 candidates showed an overlap with insignificant fluctuations (Fig. 2). Therefore, the top 75 proteins were selected as the cutoff, as the networks generated from the top 75 and 100 both retained the same overall structure (i.e., had the same degree distribution), and the top 75 network would contain less false-positive results due to the lower number of predictions.

**Fig. 3:**
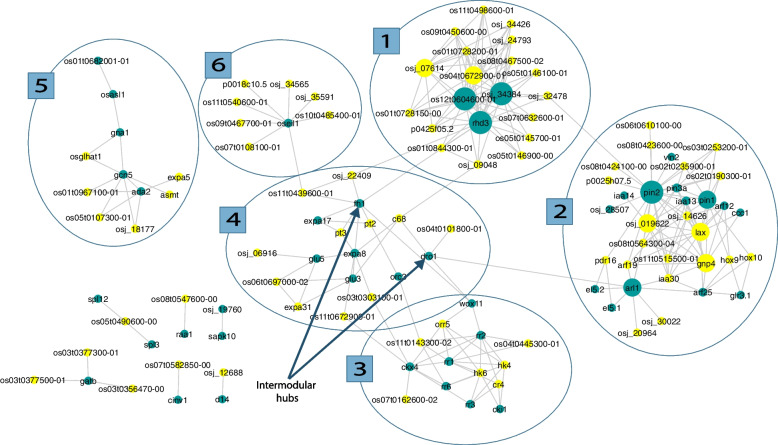
PPI network module for root development in *O. sativa* including the top 75 predicted proteins. Teal nodes represent the seed proteins, and yellow nodes represent the predicted proteins. Edges represent the interactions between proteins. The size of the node is proportional to the degree of the node. The numbered circles separate the sub-modules

Although selecting the top 100 candidates connects more proteins in the network module, visualization was not clear due to congestion, and it did not reveal novel information about new sub-modules (Supplementary Fig. 1). It was just an expansion of the existing sub-modules, which caused the integration of the 3rd and the 4th sub-modules shown in Fig. 3. Although the sub-modules 3 and 4 are integrated as in Supplementary Fig. 1, according to the enrichment analysis results, they are involved in different pathways: cytokinin-activated signaling pathway and cell wall organization, respectively. Therefore, top 75 protein candidates were selected for further analyses. Their prediction scores are given in Supplementary Table S2.

### Root development network module visualization

Visualization of the root development PPI network module with the top 75 predicted proteins is given in Fig. 3.

Altogether, there were 120 proteins including 75 predicted candidates and 45 seed proteins (Supplementary Table S2) (Fig. 3). However, 6 seed proteins (Supplementary Table S3) were not visualized in the network because 3 of those were not included in the STRING raw dataset, and 3 of those were removed while data preprocessing (Supplementary Table S3).

### Computational validation of the predictions

The enriched GO-BP terms resulting from the functional enrichment analysis of the predicted root development protein candidates are shown in Table 2. To validate their expression patterns, transcriptomic data was retrieved from the NCBI database from the project titled “Transcriptome profiling of various organs at different developmental stages in rice” (BioProject ID: PRJNA243371). The root samples for the transcriptional profiling done in the abovementioned dataset were taken before and after flowering [36].

**Table 2:** Enriched GO-BPs terms for predicted proteins that are related to root development and the pattern of expression of the corresponding genes in the root

**Term identifier**	**GO-BP term**	***P*-value**	**Proteins**	**Expression in root**
**GO:0009734**	Auxin-activated signaling pathway	0.00018	Osj_019622 (auxin response factor 16)	Broad expression before and after flowering
			Os11t0515500-01 (transport inhibitor response 1-like protein Os11g0515500)	After and before flowering
			Osj_14626 (transport inhibitor response 1-like protein Os04g0395600)	Before and after flowering
			ARF19 (auxin response factor 19-like)	Before and after flowering
			IAA30 (auxin-responsive protein IAA30-like)	Before flowering
**GO:0009736**	Cytokinin-activated signaling pathway	0.00786	HK6 (probable histidine kinase 6)	Ubiquitous expression in roots after flowering
			HK4 (probable histidine kinase 4)	Before and after flowering
			ORR5 (two-component response regulator ORR5-like)	-
			Os11t0143300-02 (two-component response regulator ORR9)	Broad expression after flowering
**GO:0071786**	Endoplasmic reticulum tubular network organization	0.00912	Os04t0672900-01 (uncharacterized protein At2g24330)	Broad expression before flowering
			Osj_07614 (uncharacterized protein At2g24330)	Ubiquitous expression in roots before flowering
**GO:0006817**	Phosphate ion transport	0.04774	PT2 (inorganic phosphate transporter 1–2) PTH-2	Biased expression in roots before flowering
			PT3 (probable inorganic phosphate transporter 1–3) PHT1-3	Expressed at low levels in roots

The GO term: auxin-activated signaling pathway (GO:0009734) is the most enriched term for the predicted protein list. Auxin is a growth coordinator hormone that regulates where, when, how much, and what sort of growth should occur in a plant. Auxins are expressed in many parts of the root such as root tip, root cap, and root epidermis. Moreover, auxins can be seen in the primary root, lateral roots, and root hairs. They are essential for many development processes in the root such as fine-tuning the growth rates, gravitropic root growth, cell division, proliferation, differentiation, and elongation of the root [37, 38].

The biological process cytokinin-activated signaling pathway (GO:0009736) was also enriched according to Table 2. Cytokinin plays several roles in root development, including regulating the responses to the growth nutrients and the biotic and abiotic stresses. Furthermore, cytokinin regulates root differentiation, elongation, branching, and root architecture [38, 39]. Moreover, it inhibits lateral root initiation and primary root elongation [39] while being essential for crown root development in *O. sativa* [4]. According to the enrichment analysis results, predicted proteins HK4, HK6, and ORR5 are in that pathway, and their network neighbors (CR4, Os11t0143300-02, Os04t0445300-01) may also have a role in root development.

Results of enrichment analysis provide strong evidence to conclude that several predicted proteins are involved in root development, and it is safe to speculate the predicted proteins as accurate predictions, which validates the prediction method.

### Identification and functional analysis of sub-modules

There were 6 identified sub-modules (Fig. 3) in the root development PPI network module. Enriched ontology terms were used to describe each sub-module. Proteins and enriched GO-BP terms for each sub-module are given below.

#### Sub-module (1)

Tables 3 and 4 contain the proteins and the enriched GO-BPs for sub-module (1). According to the results of the enrichment analysis in Table 4, it can be speculated that sub-module (1) is involved with the endoplasmic reticulum (ER) tubular network organization (GO:0071786). The ER tubular network is involved with root hair tip growth [40]. Though the hub protein RHD3 was not annotated to the ER tubular network organization (Table 4), it is involved with root hair tip growth by organizing the ER tubular network [40, 41]. Also, *Arabidopsis rhd3* mutant causes short and wavy root hairs [42]. Furthermore, HVA22d protein co-localizes with RHD3 and involves with ER shaping [41], and several proteins of the HVA22 family (protein HVA22-like protein, HVA22-like protein k, HVA22-like protein f, HVA22-like protein e, and HVA22-like protein a), which were predicted, can be seen under this sub-module. According to this information, predicted HVA22 family proteins can be speculated to be involved with root development in *O. sativa*.

**Table 3:** NCBI gene descriptions, predicted status, and hub status of the 19 proteins in sub-module (1)

**Name in network module**	**Gene description**	**Predicted status**	**Hub/non-hub**
**RHD3**	ROOT HAIR DEFECTIVE 3	Seed	Hub
**Osj_34384**	ROOT HAIR DEFECTIVE 3 Homolog 2	Seed	Hub
**Os12t0604600-01**	ROOT HAIR DEFECTIVE 3 Homolog 1	Seed	Hub
**Os04t0672900-01**	Uncharacterized protein At2g24330	Predicted	Hub
**Osj_07614**	Uncharacterized protein At2g24330	Predicted	Hub
**Os08t0467500-02**	HVA22-like protein	Predicted	Non-hub
**Os09t0450600-00**	HVA22-like protein	Predicted	Non-hub
**Osj_24793**	HVA22-like protein k	Predicted	Non-hub
**Os01s0728150-00**	HVA22-like protein f	Predicted	Non-hub
**Os11t0498600-01**	HVA22-like protein e	Predicted	Non-hub
**P0425F05.2**	HVA22-like protein a	Predicted	Non-hub
**Os05t0146900-00**	Uncharacterized	Predicted	Non-hub
**Osj_09048**	Uncharacterized	Predicted	Non-hub
**Osj_32478**	Uncharacterized	Predicted	Non-hub
**Os07t0632600-01**	Uncharacterized	Predicted	Non-hub
**Os05t0145700-01**	Uncharacterized	Predicted	Non-hub
**Os05t0146100-01**	Uncharacterized	Predicted	Non-hub
**Os01t0844300-01**	Peptidyl-prolyl cis-trans isomerase FKBP20-1	Predicted	Non-hub
**Os01t0728200-01**	Discontinues	Predicted	Non-hub

**Table 4:** Significant enriched GO-BP terms from the enrichment analysis for sub-module (1)

**Term identifier**	**Term name**	**Proteins**	***P*-value**
**GO:0000028**	Ribosomal small subunit assembly	Osj_34384, RDH3 Os12t0604600-01	0.0001
**GO:0071786**	Endoplasmic reticulum tubular network organization	Os04t0672900-01 Osj_07614	0.0018

Proteins Osj_34384, Os12t0604600-01, Os04t0672900-01, and Osj_07614 were also recognized as hubs in this sub-module. Among these, proteins Os04t0672900-01 and Osj_07614 were two predicted candidates which had not been characterized. According to the results, they probably have important roles in root development.

#### Sub-module (2)

Tables 5 and 6 contain the proteins and the enriched GO-BPs for sub-module (2), respectively.

**Table 5:** NCBI gene descriptions, predicted status, and hub status of the 34 proteins in sub-module (2)

**Name in network module**	**Gene description**	**Predicted status**	**Hub/non-hub**
**ARL1**	LOB domain-containing protein 29	Seed	Hub
**PIN1**	Auxin efflux carrier component 1	Seed	Hub
**PIN2**	Probable auxin efflux carrier Component 2	Seed	Hub
**PIN3A**	Probable auxin efflux carrier Component 3a	Seed	Hub
**IAA13**	Auxin-responsive protein IAA13	Seed	Hub
**EL5.2**	E3 ubiquitin-protein ligase EL5	Seed	Non-hub
**EL5.1**	E3 ubiquitin-protein ligase EL5	Seed	Non-hub
**ARF25**	Auxin response factor 25	Seed	Non-hub
**ARF12**	Auxin response factor 12	Seed	Non-hub
**Osj_28507**	Auxin transport protein BIG	Seed	Non-hub
**CCC1**	Cation-chloride cotransporter 1	Seed	Non-hub
**IAA14**	Auxin-responsive protein IAA14	Seed	Non-hub
**GLR3.1**	Glutamate receptor 3.1	Seed	Non-hub
**VLN2**	New submission	Seed	Non-hub
**Osj_019622**	Auxin response factor 16	Predicted	Hub
**IAA30**	Auxin-responsive protein IAA30	Predicted	Hub
**GNP4**	Protein LAX PANICLE 2	Predicted	Hub
**LAX1**	Transcription factor LAX PANICLE 1	Predicted	Hub
**Osj_14626**	Transport inhibitor response 1-like protein Os04g0395600	Predicted	Hub
**HOX10**	Homeobox-leucine zipper protein HOX10-like	Predicted	Non-hub
**HOX9**	Homeobox-leucine zipper protein HOX9	Predicted	Non-hub
**ARF19**	Auxin response factor 19	Predicted	Non-hub
**Os02t0190300-01**	Putative multidrug resistance protein	Predicted	Non-hub
**Os02t0235900-01**	Tetrahydrocannabinolic acid synthase	Predicted	Non-hub
**Os03t0253200-01**	Serine/threonine-protein kinase WAG1	Predicted	Non-hub
**Os06t0610100-00**	Discontinued	Predicted	Non-hub
**Os08t0423600-00**	Alpha carbonic anhydrase 7-like	Predicted	Non-hub
**Os08t0424100-00**	Alpha carbonic anhydrase 7	Predicted	Non-hub
**Os08t0564300-04**	ABC transporter B family member 1	Predicted	Non-hub
**Os11t0515500-01**	Transport inhibitor response 1-like protein Os11g0515500	Predicted	Non-hub
**Osj_20964**	Uncharacterized	Predicted	Non-hub
**Osj_30022**	Disease resistance protein RPM1	Predicted	Non-hub
**P0025H07.5**	Alpha carbonic anhydrase 7	Predicted	Non-hub
**PDR16**	ABC transporter G family Member 32-like	Predicted	Non-hub

**Table 6:** Selected enriched GO-BPs and KEGG pathways from the enrichment analysis for sub-module (2)

**Term Identifier**	**Term name**	**Proteins**	***P*-value**
**GO:0009734**	Auxin-activated signaling pathway	Osj_28507, Os11t0515500-01, Osj_019622, Osj_14626, PIN1, PIN2, PIN3A, IAA13, IAA30, IAA14, ARF12, ARF19, ARF25	1.914000E-19
**GO:0009926**	Auxin polar transport	Osj_28507, Os08t0564300-04, PIN3A, PIN1, PIN2	3.187130E-07
**GO:0006355**	Regulation of transcription, DNA templated	IAA14, ARF12, Osj_019622, LAX, ARF19, IAA13, HOX10, ARF25, IAA30, HOX9	0.00001
**GO:0016567**	Protein ubiquitination	Os11t0515500-01, EL5.1, Osj_14626, EL5.2	0.0002
**GO:000958**	Positive gravitropism	Os08t0423600-00, PIN2	0.0111
**osa04075 (KEGG pathway)**	Plant hormone signal transduction	IAA13, IAA14, IAA30	0.0194
**GO:0006730**	One-carbon metabolic process	Os08t0424100-00, Os08t0423600-00	0.0373
**GO:0048364**	Root development	EL5.1, EL5.2	0.0713

According to Table 6, the most enriched function for the sub-module (2) is the auxin-activated signaling pathway (GO:0009734), and most of the other functions of sub-module (2) are also related to the phytohormone auxin. The auxin signaling pathway combines transport inhibitor response 1 (TIR1), auxin response factors (ARFs), and auxin/indole acetic acid (AUX/IAA) transcriptional repressors together [38]. According to the enrichment analysis results, hub proteins Osj_019622, Osj_14626, PIN1, PIN2, PIN3A, IAA13, and IAA30 and non-hub proteins Osj_28507, Os11t0515500-01, IAA14, ARF12, ARF19, and ARF25 in sub-module (2) participate in the auxin signaling pathway.

Auxin polar transport (GO:0009926) is another enriched function for the sub-module (2) which includes PIN2, PIN1, PIN3A, Osj_28507, and Os08t0564300-04 as annotated proteins. The protein PIN2, a hub with the highest degree of 22, is a potential candidate gene for improving root system architecture in *O. sativa* [43, 44], and PIN1 and PIN3A are also hubs, which are central to the stability of this sub-module. Auxin polar transport is regulated by the PIN-FORMED (PIN) efflux carriers. PIN polarity plays a crucial role in developing proper organs and proliferation in root proximal meristem [37, 38, 45]. For example, the intramodular hub protein PIN2 works for root development by positioning and emerging root hairs [37], and according to Inahashi et al. [46], the *OsPIN2* gene regulates the seminal root elongation and lateral root formation in *O. sativa*. Moreover, overexpression of the *OsPIN2* significantly decreases the number of adventitious roots and the total root length by 22–28% [43]. Furthermore, overexpression of the gene *OsPIN3a* has led to the development of longer roots and more adventitious roots [47], and Hang et al. [47] suggest that crown root development is controlled by auxin signaling through PIN proteins. Another member of this sub-module, the protein PIN1, is also a hub protein, and overexpression of gene *OsPIN1* increases the emergence of adventitious roots, the primary root length, and the number of lateral roots [45, 48].

The roots have the ability to change their growing orientation in response to the changes in gravity [37, 44], and it is controlled by the asymmetric distribution of auxin at the root tip. PIN family, AUX1 (AUXIN-INSENSITIVE1), and other members of the auxin transport pathway contribute to this auxin distribution [37, 44]. Deletion in the *OsPIN2* gene has displayed gravitropic root growth phenotypes as shootward auxin distribution in the lower side of the root is largely repressed during a gravity stimulus by the mutation of *OsPIN2* [43, 44]. This shows that PIN2 is also essential for root gravitropism. Moreover, the crown root growth angle is an important component of the *O. sativa* roots, and the *OsPIN2* mutant, *lra1*, has displayed larger root angles [44]. This indicates that the protein PIN2 is important in regulating crown root growth angle. Furthermore, *OsPIN3a* has shown a notable upregulation in *OsPIN2* mutant *lra1* since *OsPIN3a* compensates for the loss of *OsPIN2* (agravitropic root phenotype) to some extent [44]. One of the enriched terms for the sub-module (2) is positive gravitropism (GO:0009958), and the seed protein PIN2 and the predicted candidate Os08t0423600-00 have been annotated to that process. In conclusion, this evidence prove that the majority of the sub-module 2 proteins contribute to root development via auxin regulation.

#### Sub-module (3)

Tables 7 and 8 contain the proteins and the enriched GO-BPs for sub-module (3), respectively. According to Table 8, the top enriched GO-BP term for sub-module (3), cytokinin-activated signaling pathway (GO:0009736), is mediated by a two-component system, and the signaling is transmitted by transcription activators and repressors in a phosphorylation signal transduction system (GO:0000160) [49, 50]. The two-component system comprises three functional modules: sensory histidine kinase (HK), histidine phosphate transfer protein (HP), and response regulator (RR). Cytokinins are sensed by membrane-located HK receptors that transmit signals via HPs to nuclear RRs that activate or repress transcription [51].

**Table 7:** NCBI gene descriptions, predicted status, and hub status for the 14 proteins in sub-module (3)

**Name in network module**	**Gene description**	**Predicted status**	**Hub/non-hub**
**RR1**	Two-component response regulator ORR1	Seed	Hub
**RR2**	Two-component response regulator ORR2	Seed	Hub
**CKX4**	Cytokinin dehydrogenase 4	Seed	Hub
**RR3**	Two-component response regulator ORR3	Seed	Non-hub
**RR6**	Two-component response regulator ORR6	Seed	Non-hub
**CKI1**	Casein kinase 1	seed	Non-hub
**WOX11**	WUSCHEL-related homeobox 11	Seed	Non-hub
**CR4**	Putative receptor protein kinase CRINKLY4	Predicted	Non-hub
**HK4**	Probable histidine kinase 4	Predicted	Non-hub
**HK6**	Probable histidine kinase 6	Predicted	Non-hub
**ORR5**	Two-component response Regulator ORR6	Predicted	Non-hub
**Os11t0143300-02**	Two-component response regulator ORR9	Predicted	Non-hub
**Os04t0445300-01**	Uncharacterized	Predicted	Non-hub
**Os07t0162600-02**	Probable carboxylesterase	Predicted	Non-hub

**Table 8:** Selected enriched GO-BPs and KEGG pathways of enrichment analysis for sub-module (3)

**Term identifier**	**Term name**	**Protein**	***P*-value**
**GO:0009736**	Cytokinin-activated signaling pathway	HK4, HK6, RR2, RR6, RR1, RR3, ORR5	1.166273E-12
**GO:0000160**	Phosphorylation signal transduction system	RR2, RR6, RR1, RR3, ORR5	5.924492E-08
**osa04075(KEGG pathway)**	Plant hormone signal transduction	RR2, RR6, RR1, RR3, ORR5	0.00002
**GO:0006355**	Regulation of transcription, DNA templated	RR2, RR6, RR1, RR3, ORR5, WOX11	0.00045
**GO:0006468**	Protein phosphorylation	HK4, HK6, CR4	0.0163

The phytohormone cytokinin is present in the *O. sativa* root [43] and participates in regulating root development and root architecture. This indicates the involvement of the cytokinin-activated signaling pathway in *O. sativa* root development and the involvement of sub-module (3) proteins in root development through the cytokinin-activated signaling transduction pathway.

#### Sub-module (4)

Tables 9 and 10 contain the proteins and the enriched GO-BPs in sub-module (4).

**Table 9:** NCBI gene descriptions, predicted status, and hub status for the 18 proteins in sub-module (4)

**Name in network module**	**Gene description**	**Predicted status**	**Hub/non-hub**
**FH1**	Formin-like protein 1	Seed	Hub
**DRO1**	Uncharacterized	Seed	Hub
**GLU3**	Endoglucanase 12	Seed	Non-hub
**GLU5**	Endoglucanase 2	Seed	Non-hub
**ORC3**	Origin of replication complex subunit 3	Seed	Non-hub
**EXPA8**	Expansin-A8	Seed	Non-hub
**EXPA17**	Expansin-A17-like	Seed	Non-hub
**C68**	Probable LRR receptor-like Serine/threonine-protein kinase At5g45780	Predicted	Non-hub
**EXPA31**	Expansin-A31	Predicted	Non-hub
**Os03t0303100-01**	Serine/arginine repetitive matrix protein 2	Predicted	Non-hub
**Os04t0101800-01**	Uncharacterized	Predicted	Non-hub
**Os06t0697000-02**	Probable xyloglucan Endotransglucosylase/hydrolase protein 25	Predicted	Non-hub
**Os11t0439600-01**	Probable apyrase 3	Predicted	Non-hub
**Os11t0672900-01**	Serine/arginine repetitive matrix protein 1	Predicted	Non-hub
**Osj_06916**	Uncharacterized	Predicted	Non-hub
**Osj_22409**	COBRA-like protein 10	Predicted	Non-hub
**PT2**	Inorganic phosphate transporter 1–2	Predicted	Non-hub
**PT3**	Probable inorganic phosphate transporter 1–3	Predicted	Non-hub

**Table 10:** Selected enriched GO-BPs of enrichment analysis for sub-module (4)

**Term identifier**	**Term name**	**Proteins**	***P*-value**
**GO:0071555**	Cell wall organization	GLU5, GLU3, Os06t0697000-02	0.0091
**GO:0006817**	Phosphate ion transport	PT2, PT3	0.0129
**GO:0030245**	Cellulose catabolic process	GLU5, GLU3	0.0185

As given in Table 10, sub-module (4) has proteins that are associated with both plant cell wall organization (GO:0071555) and cellulose catabolic process (GO:0030245) pathways. The proteins GLU5, GLU3, and Os06t0697000-02 are involved with cell wall organization, and GLU5 and GLU3 are involved with cellulose catabolic process. Cell walls are important in any form of plant development, and cellulose is the major component of the plant cell wall. Cellulose biosynthesis and cell wall loosening enable turgor-driven cell expansion in growing plants, and it has been speculated that endo-1,4-b-glucanases (EGases) play a central role in these complex activities [52]. GLU3 and GLU5 (GLUs) have been directly annotated to the hydrolysis of endo-1,4-b-glucanases [53, 54]. GLUs play important roles in root development, and *glu* mutants have reduced root development [54]. Furthermore, GLU5 is expressed in lateral root primordia during auxin-induced lateral root development [53].

Phosphate ion transport (GO:0006817) in a plant is mediated by several transporter protein families such as the Pht1 family [55]. PT2 and PT3 belong to the pht1 family and are predicted candidates in sub-module (4). Both root hair length and frequency increase in response to phosphate (Pi) starvation, and the gene expression of *OsPT2* is increased during Pi starvation. Therefore, it is reasonable to speculate that PT2, which is a predicted candidate protein, probably has a direct association with Pi transport [55, 56].

FH1 is an intramodular hub in sub-module (4) and also an intermodular hub. FH1 has been directly annotated to root hair development [57, 58]. EXPA8 in sub-module 4 (degree = 7) was not considered a hub according to the hub selection criteria of this study. However, it is a root-specific expansin protein, and expansins are plant cell wall proteins that are involved in cell wall modifications [59, 60]. Overexpression of the *OsEXPA8* gene has shown improved root system architecture with longer primary roots and a higher number of lateral roots and root hairs [59, 60]. Moreover, repression of *OsEXPA8* has reduced the cell size of the root vascular system and plant height [60]. This evidence proves that sub-module (4) is linked to root development via cell wall organization.

Proteins of other sub-modules are listed in Supplementary Table S4. They did not have any significant enriched GO-BP terms related to root development and need further investigation to confirm their involvement in root development.

### Identification and analysis of hub proteins

There are two types of hubs: intramodular and intermodular hubs [22]. Intramodular hubs are found within a functional module, while intermodular hubs act between the modules to interconnect them [21–23].

#### Intramodular hubs

For this study, the top 10% of proteins with the highest degrees were selected [61] as intramodular hubs, which corresponds to a degree cutoff of 8, resulting in 20 proteins (Table 11).

**Table 11:** Details of intramodular hub proteins

**Protein**	**Degree**	**Type (seed/predicted)**
**PIN2**	22	Seed
**RHD3**	19	Seed
**Osj_34384**	17	Seed
**Os12T0604600-01**	17	Seed
**LAX1**	13	Predicted
**GNP4**	12	Predicted
**Os04T0672900-01**	11	Predicted
**Osj_07614**	11	Predicted
**Osj_019622**	10	Predicted
**ARL1**	10	Seed
**PIN1**	10	Seed
**PIN3A**	9	Seed
**IAA30**	8	Predicted
**Osj_14626**	8	Predicted
**FH1**	8	Seed
**IAA13**	8	Seed
**CKX4**	8	Seed
**RR2**	8	Seed
**RR1**	8	Seed
**OSEIL1**	8	Seed

Some of the hubs are from the seed proteins, and others are predicted candidates for root development in *O. sativa*. The predicted hubs are important findings because their relevance in root development has not been revealed to date. They also confirm the accuracy and importance of the predictions. Among the predicted hubs, there were candidates annotated to the enriched GO-BP terms related to the root development in *O. sativa*, although they lack direct experimental evidence for root development. For example, *Os04t0672900-01* and *Osj_07614* of sub-module (1) were annotated to GO:0071786: endoplasmic reticulum tubular network organization (Table 4), which is a GO-BP term associated with root development.

Moreover, transcription factors LAX1 (LAX PANICLE 1) and GNP4 (LAX PANICLE 2) are predicted candidates, which were identified as hubs. In *O. sativa*, LAX1 and GNP4 are required for the formation of axillary meristem throughout the plant’s lifespan [62, 63]. Also, LAX1 shows non-cell-autonomous action (mutant extends beyond the mutant cells); however, its molecular basis has not been revealed yet [62]. Although the functions of lax genes in *O. sativa* panicle have been studied, their functions in the root are yet to be revealed. The above results provide evidence for their involvement in root development. Therefore, this study provides potential candidates for selecting important proteins for future *O. sativa* root development studies.

The seed protein OSEIL1 is an intramodular hub and is involved in root development in *O. sativa*. It is a transcription factor participating in the ethylene signaling pathway, which promotes *O. sativa* root elongation [64]. Most importantly, OSEIL1 connects with 8 predicted candidates and joins the sub-modules (4) and (6) together (Fig. 3). Therefore, according to our results, OSEIL1 is a critical protein for root development and a likely candidate for future genetic engineering studies.

#### Intermodular hubs

Intermodular hubs connect different sub-modules (Fig. 3) and are important in linking the different metabolic/biological pathways. Two intermodular hubs were detected in this study (Table 12).

**Table 12:** Details of intermodular hub proteins

**Intermodular hub**	**Degree**	**Type (seed/predicted)**	**Connected proteins**	**Sub-module (**Fig. 3**)**
**DRO1 (in 4th sub-module)**	*4*	Seed	C68 Os04t0101800-01	4
			ARL1	2
			WOX11	3
**FH1 (in 4**th** sub-module)**	*8*	Seed	PT3, PT2, EXPA8, EXPA17, Osj_22409, Os11t0439600-01	4
			RHD3	1
			WOX11	3

As shown in Table 12, the protein DRO1 (DEEPER ROOTING 1) in sub-module 4 works as an intermodular hub and connects sub-modules 2, 3, and 4. Therefore, disturbance to the DRO1 can potentially disrupt the interconnectivity of the pathways or the proper mechanism of those sub-modules. Analysis of DRO1 using iDNA-Prot (http://www.jci-bioinfo.cn/iDNA-Prot) [65], which is a web tool for identifying DNA binding domains in proteins [65], revealed that it may be a DNA-binding protein. Highly expressed *DRO1* gene is involved in the regulation of deep rooting by increasing root growth angle which promotes the root growth in a more downward direction [66]. Furthermore, *DRO1* enhances nitrogen uptake and cytokinin fluxes at late stages of development by deep rooting which resulted in a high yield in *O. sativa*. Therefore, *DRO1* can be used to develop *O. sativa* cultivars that have high yields under both drought and non-drought conditions by controlling the root system architecture [66, 67]. As shown in this study, the intermodular hub DRO1 plays a major role in interconnecting and potentially regulating the 3 submodules of root development and can be a valuable candidate for further experimental studies.

The FH1 (formin-like protein 1), which is in the sub-module (4), is an intramodular hub and a critical regulator of the *O. sativa* root hair development. *Osfh1* mutant exhibited growth defects on root hairs. These defects depend on the environmental conditions and were only exhibited when roots were submerged in a solution [57]. According to Huang et al. [58], the external supplies of nutrients or hormones could not rescue the defective mutant. Therefore, FH1 is a crucial protein for the growth of *O. sativa* as rice is grown under water-logged conditions in the field until the fruit ripening stage [57, 58]. FH1 is also identified as an intermodular hub, and it connects sub-modules (1), (3), and (4) (Table 12, Fig. 3). Formins regulate the growth and elongation of the root hairs and cell wall loosening and synthesis, which are required for root hair development [57]. Furthermore, the sub-module (4) which is annotated to the cell wall organization and the sub-module (1) which is mainly recognized for root hair development are connected by FH1. Since it is connected with 3 sub-modules, it could have more roles in different pathways which are not yet revealed.

## Discussion

The large number of proteins interacting with each other during the development of various plant systems makes disentangling their roles using traditional experimental techniques a daunting task. Computational approaches are ideal here as they enable the aggregation of data to construct higher-level views of biological systems (such as PPI network graphs), which are much better at identifying complex relationships between proteins.

During this study, we predicted 75 novel protein candidates associated with root development. Validation of these predictions and analysis of hubs and sub-modules justified that some predictions are annotated to biological processes associated with root development, which confirmed the accuracy of the predictions. These predictions are based on network-based candidate gene prediction, which has been proven to be an accurate method [15]. We also predicted 20 intramodular hubs and 2 intermodular hubs, which are important outcomes of this study. This enabled identifying the important module members central to the stability of the root development in *O. sativa*. These hub proteins are potential candidates for future genetic engineering experiments as their influence on root development is larger than other proteins because of their centralized nature. The association of several identified hub proteins, such as PIN2 and DRO1, with root development is already experimentally validated. However, there are predicted hub proteins, such as LAX1 and GNP4, which require further experimental investigation. Therefore, this study provides a plethora of protein candidates for future experiments.

Importantly, this study depicts the organization of PPI interactions underlying root development. We unraveled how proteins associated with root development are organized into 6 major sub-modules, mainly attributed to biological processes, such as ER tubular network organization, auxin regulation pathway, cytokinin signaling pathway, and cell wall synthesis. The knowledge about molecular mechanisms and properties of a majority of these module proteins are still incomplete; hence, this analysis provides clues to their collective role in regulating root development in *O. sativa*.

## Conclusion

In this study, we analyzed the network structure of root development proteins, during which 75 new protein candidates, 6 sub-modules, 20 intramodular hubs, and 2 intermodular hubs were identified using a computational analysis. This opens up new directions for future wet lab and dry lab studies based on predicted candidates and hub proteins. To our knowledge, this is the first study that analyzes the PPI network module for root development in *O. sativa*. Therefore, these findings are the first to show the PPI interaction structure underlying root development, which depicts the importance and applicability of network analysis on other plant developmental phenotypes as well.

## Supplementary Information

**Additional file 1: Supplementary Table S1.** Details of seed proteins. **Supplementary Table S2.** List of NCBI gene symbols of proteins of extracted network module and the prediction scores for the top 75 candidates. **Supplementary Table S3.** Details of missing seed proteins. **Supplementary Table S4.** Details of sub-modules.

**Additional file 2: Supplementary Fig. 1.** PPI network module visualization with 100 predicted proteins and 45 seed proteins.

## Data Availability

All data generated or analyzed during this study are included in this published article and in its accompanying supplementary information files. Python scripts written for this analysis are available at https://github.com/Samadhi9/PPIN-analysis.

## Abbreviations

PPI: Protein-protein interactions
IAA: Indole-3-acetic acid
NAA: Naphthaleneacetic acid
IDs: Identifiers
GO: Gene Ontology
GO-BP: Biological process component of the Gene Ontology
ER: Endoplasmic reticulum
PIN: PIN-FORMED
HK: Histidine kinase
HP: Histidine phosphor transfer
RR: Response regulator
Pi: Phosphate
FH2: Formin homology-2
PR: Primary root
CR: Crown root
ARF: Auxin response factor
EGases: Endo-1,4-b-glucanase
