# Supplementary material for: Protein-protein interaction (PPI) network analysis reveals important hub proteins and sub-network modules for root development in rice (Oryza sativa)
Source: J Genet Eng Biotechnol. 2023 May 29;21:69. doi: 10.1186/s43141-023-00515-8 (PMC10225403; doi:10.1186/s43141-023-00515-8)
Supplement: Supplementary file 1 — Additional file 1: Supplementary Table S1. Details of seed proteins. Supplementary Table S2. List of NCBI gene symbols of proteins of extracted network module and the prediction scores for the top 75 candidates. Supplementary Table S3. Details of missing seed proteins. Supplementary Table S4. Details of sub-modules. [file 43141_2023_515_MOESM1_ESM.pdf]

Additional file 1

Tables

Supplementary Table S1: Details of seed proteins

Protein	Specific location	Involvement in root development	Function	Source
**ada2**	Primary root (PR), crown root (CR)	Root elongation of PR and CR, increase number of CR	Auxin or cytokinin signaling	Literature[1]
**aim1**	Root meristem	Root growth	Salicylic acid biosynthesis promotes reactive oxygen species accumulation	Literature[2]
**arf1**	Adventitious root	Root formation	Auxin response factor, transcriptional factor	Literature [3]
**arf12**	Primary root	Longer primary roots	Auxin response factor, transcriptional factor	Literature [3,4]
**arf25**	Crown root	Root development	Auxin response factor, transcriptional factor	Literature[4]
**arl1**	Crown root, lateral root, adventitious root	Root formation, root development	Auxin-mediated cell de-differentiation	Literature [3]
**crl4/gnom1**	Crown root	Root initiation	Polar auxin transportation	Literature[5]
**ccc1**	Root tip	Root elongation	Osmotic regulation	Literature[6]
**cinv1**	Primary root, lateral root	Root elongation, root formation	Cleaves sucrose into glucose and fructose, floral transition, and pollen development	STRING DB
**cki1**	Lateral root, adventitious root, primary root	Root elongation, increase the number of roots.	Involved in ABA and brassinosteroid signaling pathways.	STRING DB

**ckx4**	Crown root	Root formation, root emergence, and development increase the number of crown roots.	CK signaling pathway	Literature[7]
**cinv1 (cyt-inv1)**	-	Root elongation	Mutant accumulates sucrose and had reduced levels of hexose	Literature[8]
**d14**	Crown roots	Root elongation	Involved in the strigolactone signaling pathway	Literature [9]
**DRO1**	Crown roots	Increases root angle and facilitate root growth more downward	enhances nitrogen uptake and cytokinin fluxes	Literature [10]
**el5.1/el5.2**	Root primordia	Maintains cell viability after the initiation of root primordial formation,	mediate the degradation of cytotoxic proteins produced in root cells	STRING DB
**expa17**	Root hair	Root elongation	Cell wall remodeling	Literature[11]
**expa8**	Root and shoot	Root elongation, fewer LRs, and short root hairs	Encodes for cell wall localized protein	Literature[11]
**fh1**	Root hair	Root elongation	Actin-binding protein	Literature[12]
**gatb**	Primary root, root tip	Growth, cell division, and elongation	Maintaining mitochondrial structure and function	Literature[13]
**gcn5**	Primary root, crown root	Root elongation of PR and CR, increase the number of CR	Auxin or cytokinin signaling	Literature[1]
**glr3.1**	Root meristem (apical)	Root elongation, maintenance of cell division, survival in	Glutamate-gate receptor	Literature[14]

		the root meristem in early seedlings		
**glu3**	-	Root elongation	Encodes for putative membrane-bound endo-1,4-β-glucans. Mutant lower the cellulose contents in root cell walls	Literature[9]
**glu5**	Lateral root development	Root development	Endohydrolysis of (1->4)-beta-D-glucosidic linkages in cellulose	STRING DB
**gna1**	Root elongation zone	Root elongation, maintaining normal root cell shape	Plays an important role in protein and lipid glycosylation	Literature[15]
**iaa13**	Lateral root	Root formation	Member of IAA gene family, transcriptional factor	Literature[16]
**iaa14**	Lateral root	Root initiation	Member of IAA gene family, transcriptional factor	Literature[17]
**mst3**	Root system	Root development	Sugar transporter	STRING DB
**mt2c**	Lateral root	Root development, root initiation	Seed embryo germination by regulating CK level, reactive oxygen species scavenger in the cytosol	STRING DB
**orc3**	Lateral root	Root development	Component of the origin recognition complex that binds origins of replication	Literature[3]
**os01t0682001-01**	Seedling roots	Primary ammonium ions assimilation	Reutilization of glutamine in developing organs.	STRING DB

			Plays a role in the development of tillers	
**os12t0604600-01**	Root hair	Root development	Probable GTP-binding protein	STRING DB
**osasl1**	Primary root, root	Primary root elongation, normal root growth	Arginine biosynthesis	Literature[18]
**oseil1**	-	Root elongation	Involved in the ethylene signaling pathway, transcriptional factor	Literature[19]
**osj_19760**	Root hair	Form shorter root hairs	Reduced ABA signaling	Literature[20]
**osj_28507**	Lateral root	Influencing auxin-mediated developmental responses root production (ex: cell elongation, apical dominance, general growth, and development)	Auxin efflux and polar auxin transport	STRING DB
**osj_34384**	Root hair	Root development	Probable GTP-binding protein	STRING DB
**pin1**	adventitious root	Regulation of adventitious root development via auxin pathway	Controls the traffic of auxin efflux carrier proteins	Literature[3,21]
**pin2**		Root growth angle	Component of the auxin efflux carrier	Literature[3,21]
**pin3a**	Crown root	Root development, response to water stress	Auxin efflux and polar auxin transport	STRING DB

**raa1**	Root system	Root development mediate by auxin	Cell cycle regulator during root development	STRING DB
**rcn1**	Lateral root	Promoting the outgrowth, hypodermal suberization of roots	Essential transporter for growth and development under abiotic stress, required for salt tolerance via Na/K homeostasis	STRING DB
**rhd3**	Root hair	Root development	Probable GTP-binding protein	Literature[12,22–24]
**rr1**	Crown root	Root development	CK response regulators	Literature[21,25–30]
**rr2**	Crown root	Root development	CK response regulators	Literature [21,25,26,28,30]
**rr3**	Crown root	Root development	CK response regulators	Literature[26,30]
**rr6**	Crown root	Suppressed root and vegetative development	CK response regulators	Literature[26,30]
**sapk10**	Root hair	Produces longer root hairs	Aba activated protein kinase 10 production	Literature[31]
**spl12**	Crown roots	Root development	Trans-acting factor	Literature[32]
**spl3**	Crown roots	Increase number of roots	Trans-acting factor	Literature[32]
**vln2**		Root gravitropism	Modulation of polar auxin transport	Literature[33]
**wox11**	Crown root	Root elongation, root development	CK-regulation	Literature[1,34]

Supplementary Table S2:

List of NCBI gene symbols of proteins of extracted network module and the prediction scores for the top 75 candidates

	Protein	Gene symbol	Type of protein (seed/predicted)	Prediction score
**1**	Os07t0108100-01	LOC4342208	Predicted	398.2289137
**2**	Osj_06916	LOC4329481	Predicted	398.2289137
**3**	Os05t0145700-01	LOC4337795	Predicted	394.2489024
**4**	Os05t0146100-01	LOC4337797	Predicted	394.2489024
**5**	Os05t0146900-00	LOC9272414	Predicted	394.2489024
**6**	GNP4	LOC107277161	Predicted	278.8703058
**7**	PDR16	LOC4326812	Predicted	271.1123074
**8**	LAX	LOC4327431	Predicted	262.3899959
**9**	EXPA5	LOC4330706	Predicted	198.1182047
**10**	P0018C10.5	LOC4325089	Predicted	198.1182047
**11**	Os11t0540600-01	LOC4350668	Predicted	198.1182047
**12**	Os06t0610100-00	Discontinued	Predicted	198.1182047
**13**	Os08t0423600-00	LOC107276201	Predicted	198.1182047
**14**	Osj_18177	LOC 4338502	Predicted	196.1331962
**15**	Osj_07614	LOC4330055	Predicted	194.1581821
**16**	Os11t0143300-02	LOC4349747	Predicted	192.1931623
**17**	HK6	LOC107275680	Predicted	175.4249306
**18**	C68	LOC4328829	Predicted	174.1518585
**19**	Os04t0672900-01	LOC4337370	Predicted	174.1518585
**20**	Os08t0467500-02	LOC4345793	Predicted	174.1518585
**21**	Os03t0253200-01	LOC4332276	Predicted	174.1518585
**22**	Os05t0107300-01	LOC107275998	Predicted	173.900894
**23**	P0425F05.2	LOC4340552	Predicted	165.5780768
**24**	Osj_34426	LOC4350866	Predicted	157.7839569
**25**	Os02t0190300-01	LOC4328570	Predicted	157.7839569
**26**	Os01t0967100-01	LOC4324005	Predicted	156.1155519
**27**	Osj_24793	LOC4343642	Predicted	150.6678038
**28**	HK4	LOC4333916	Predicted	146.4983529
**29**	Osj_09048	LOC4331278	Predicted	144.1448717
**30**	HOX9	LOC4348919	Predicted	141.028896
**31**	Os09t0450600-00	LOC4347222	Predicted	138.1439741
**32**	Os01t0728200-01	Discontinued	Predicted	138.1439741
**33**	Os07t0632600-01	LOC4344004	Predicted	132.6048761

**34**	Os07t0162600-02	LOC4342462	Predicted	131.4163008
**35**	Os03t0356470-00	LOC9270588	Predicted	131.4163008
**36**	Os03t0377300-01	LOC9267254	Predicted	131.4163008
**37**	Os03t0377500-01	LOC9271339	Predicted	131.4163008
**38**	Osj_34565	LOC4350998	Predicted	131.4163008
**39**	Os10t0485400-01	Discontinued	Predicted	131.4163008
**40**	Os08t0547600-00	LOC9269394	Predicted	131.4163008
**41**	Osj_12688	Discontinued	Predicted	131.4163008
**42**	Os08t0424100-00	LOC9267362	Predicted	131.4163008
**43**	P0025H07.5	LOC4347247	Predicted	131.4163008
**44**	EXPA31	LOC4333168	Predicted	129.4387881
**45**	Os11t0515500-01	LOC4350590	Predicted	122.7141653
**46**	PT2	LOC4331637	Predicted	122.7141653
**47**	Os01t0728150-00	LOC107276471	Predicted	118.2806568
**48**	PT3	LOC4348740	Predicted	118.2806568
**49**	Osj_32478	LOC4349400	Predicted	115.2765157
**50**	ARF19	LOC4341978	Predicted	103.235112
**51**	ORR5	LOC4336439	Predicted	103.235112
**52**	Os04t0445300-01	LOC4335958	Predicted	102.7645228
**53**	Os06t0697000-02	LOC4341944	Predicted	102.7645228
**54**	Os07t0582850-00	LOC9266807	Predicted	98.06659812
**55**	Os04t0101800-01	LOC4334888	Predicted	98.06659812
**56**	Osj_35591	Discontinued	Predicted	98.06659812
**57**	Os09t0467700-01	LOC4347323	Predicted	98.06659812
**58**	CR4	LOC4333525	Predicted	92.21651326
**59**	Osj_22409	LOC4341879	Predicted	90.21339716
**60**	Os08t0564300-04	LOC4346344	Predicted	89.18949213
**61**	Os11t0498600-01	LOC4350556	Predicted	88.88541299
**62**	Os02t0235900-01	LOC9271032	Predicted	86.45738679
**63**	Osj_019622	LOC9270361	Predicted	85.55435509
**64**	Os11t0439600-01	LOC4350420	Predicted	84.9841779
**65**	Os11t0672900-01	LOC4351100	Predicted	84.9841779
**66**	Os03t0303100-01	LOC4332586	Predicted	84.9841779
**67**	Osj_14626	LOC4335696	Predicted	84.15088682
**68**	Os01t0844300-01	LOC4327507	Predicted	81.95702094

**69**	Os05t0490600-00	LOC4339167	Predicted	80.30566578
**70**	OsGLHAT1	LOC107275998	Predicted	80.30566578
**71**	HOX10	LOC4331345	Predicted	79.86774383
**72**	IAA30	LOC4352721	Predicted	79.86774383
**73**	ASMT	LOC4346795	Predicted	78.05777595
**74**	Osj_20964	LOC4340753	Predicted	78.05777595
**75**	Osj_30022	LOC4347587	Predicted	78.05777595
**76**	ADA2	LOC4334126	Seed	-
**77**	ARF12	LOC4337363	Seed	-
**78**	ARF25	LOC4352783	Seed	-
**79**	ARL1	LOC9271993	Seed	-
**80**	CCC1	LOC4345272	Seed	-
**81**	CINV1	LOC4329626	Seed	-
**82**	CKI1	LOC4330018	Seed	-
**83**	CKX4	LOC4326515	Seed	-
**84**	D14	LOC4331983	Seed	-
**85**	DRO1	LOC4347169	Seed	-
**86**	EL5.1	LOC107276751	Seed	-
**87**	EL5.2	LOC4329685	Seed	-
**88**	EXPA17	LOC107276467	Seed	-
**89**	EXPA8	LOC4327217	Seed	-
**90**	FH1	LOC4325107	Seed	-
**91**	GATB	LOC4350686	Seed	-
**92**	GCN5	LOC4348629	Seed	-
**93**	GLR3.1	LOC4336790	Seed	-
**94**	GLU3	LOC4336284	Seed	-
**95**	GLU5	LOC4324643	Seed	-
**96**	GNA1	LOC4347432	Seed	-
**97**	IAA13	LOC4334069	Seed	-
**98**	IAA14	LOC4334431	Seed	-
**99**	MST3	LOC4342198	Seed	-
**100**	MT2C	LOC4337596	Seed	-
**101**	ORC3	LOC4324643	Seed	-
**102**	Os01t0682001-01	LOC4324398	Seed	-
**103**	Os12t0604600-01	LOC4352733	Seed	-

**104**	OsASL1	LOC4332596	Seed	-
**105**	OsEIL1	LOC4332697	Seed	-
**106**	Ost_28507	LOC4346497	Seed	-
**107**	Osj_34384	LOC4350788	Seed	-
**108**	PIN1	LOC4330700	Seed	-
**109**	PIN2	LOC4341736	Seed	-
**110**	PIN3A	LOC4326565	Seed	-
**111**	RAA1	LOC4326229	Seed	-
**112**	RCN1	LOC4332449	Seed	-
**113**	RHD3	LOC4326314	Seed	-
**114**	RR1	LOC4335937	Seed	-
**115**	RR2	LOC4329677	Seed	-
**116**	RR3	LOC4331245	Seed	-
**117**	RR6	LOC4337372	Seed	-
**118**	SAPK10	LOC4333435	Seed	-
**119**	VLN2	New submission	Seed	-
**120**	WOX11	LOC4344325	Seed	-

Supplementary Table S3: Details of missing seed proteins

**Absent in raw dataset**	**Removed by filtering**
AIM1	Osj_19760
CARK	HO1
SPL12	SPL3

Supplementary Table S4: Details of sub-modules

SUB-MODULE	SEEDS	Functions of seeds	PREDICTED CANDIDATES
1<br>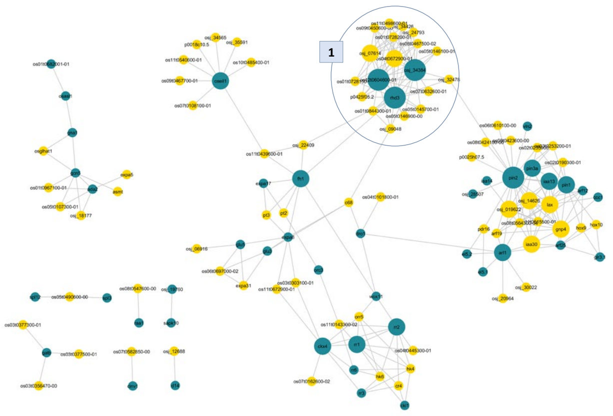	RHD3<br>Osj_34384<br>Os12t0604600-01	Root hair development	Osj_32478<br>Osj_07614<br>Osj_24793<br>P0425F05.2<br>Os01t0728200-01<br>Os07t0632600-01<br>Os05t0146900-00<br>Osj_09048<br>Os09t0450600-00<br>Os01t0728150-00<br>Os05t0145700-01<br>Os04t0672900-01<br>Os05t0146100-01<br>Os08t0467500-02<br>Osj_34384<br>Os01t0844300-01<br>Os11t0498600-01

2

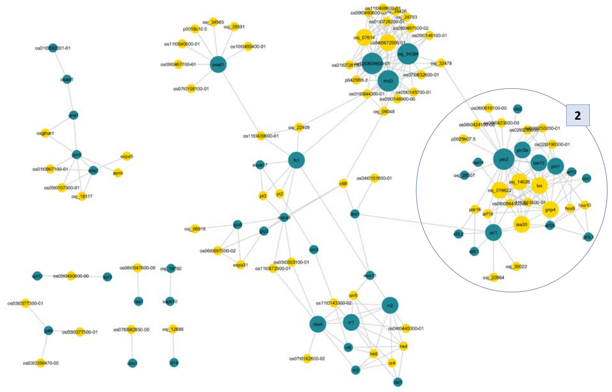

EL5.2<br>EL5.1	Degradation of cytotoxic proteins produced in root cells
ARF25<br>ARF12	Auxin response factors
Osj_28507	Influence in auxin-mediated developmental responses
CCC1	Osmotic regulation
ARL1	Auxin-mediated cell dedifferentiation
PIN3A<br>PIN2<br>PIN1	Auxin efflux and polar auxin transport
IAA13<br>IAA14	Members of the IAA family
VLN2	Modulation of polar auxin transport
GLR3.1	The glutamate-gate receptor may regulate cell proliferation and cell death in the root apex

ARF19
GNP4
HOX10
HOX9
IAA30
LAX
Os02t0190300-01
Os02t0235900-01
Os03t0253200-01
Os06t0610100-00
Os08t0423600-00
Os08t0424100-00
Os08t0564300-04
Os11t0515500-01
Osj_019622
Osj_14626
Osj_20964
Osj_30022
P0025H07.5
PDR16

**3** 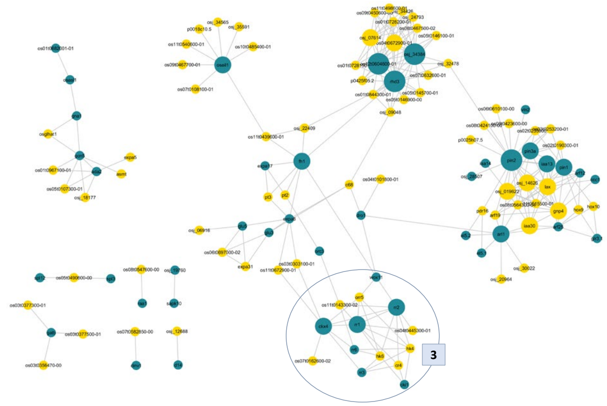	CKI1	Involved in ABA and brassinosteroid signaling pathways.	CR4<br>HK4<br>HK6<br>ORR5<br>Os04t0445300-01<br>Os07t0162600-02<br>Os11t0143300-02
	RR6<br>RR1<br>RR2<br>RR3	CK response regulators	
	CKX4	CK signaling pathway	
	WOX11	CK-regulation	
**4** 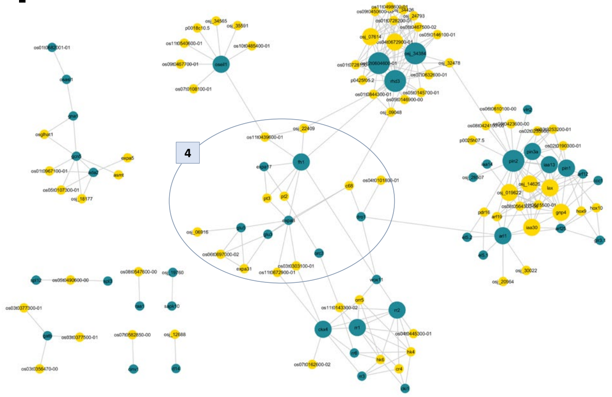	FH1	Root hair development	C68<br>EXPA31<br>Os03t0303100-01<br>Os04t0101800-01<br>Os06t0697000-02<br>Os11t0439600-01<br>Os11t0672900-01<br>Osj_06916<br>Osj_22409
	GLU3	Encoded for putative membrane-bound<br>Endo-1,4-β-glucanase, mutant lower the cellulose contents in its root cell walls	
	GLU5	Endohydrolysis of (1->4)-beta-D-glucosidic linkages in cellulose	

	ORC3	Component of the origin recognition complex that binds origins of replication	PT2<br>PT3
	EXPA8	Cell wall localized protein	
	EXPA17	Cell wall remodeling	
	DRO1	Enhances nitrogen uptake and cytokinin fluxes, regulating root growth angle	
**5**<br>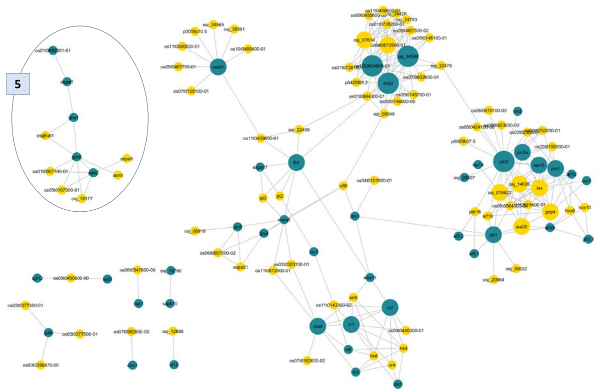	GNA1	Plays an important role in protein and lipid glycosylation	ASMT<br>EXPA5<br>Os01t0967100-01<br>Os05t0107300-01<br>OsGLHAT1<br>Osj_18177
	ADA2	Auxin or cytokinin signaling	
	Os01t0682001-01	Reutilization of glutamine in developing organs. Plays a role in the development of tillers	
	gcn5	Auxin and cytokinin signaling	
	osasl1	Arginine biosynthesis	

**6** 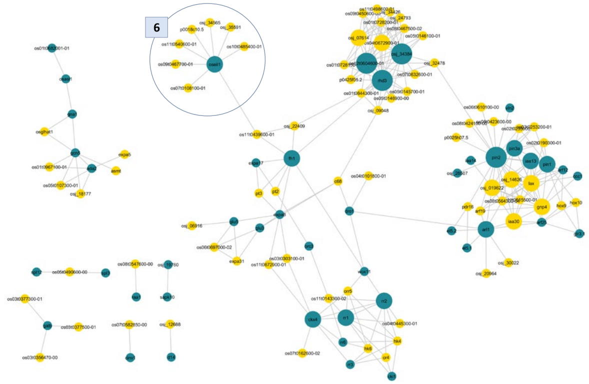	oseil1	Involved in the ethylene signaling pathway	Os07t0108100-01<br>Os09t0467700-01<br>Os10t0485400-01<br>Os11t0540600-01<br>OSj_34565<br>Osj_35591<br>P0018C10.5

References

[1] Zhou S, Jiang W, Long F, Cheng S, Yang W, Zhao Y, et al. Rice Homeodomain Protein WOX11 Recruits a Histone Acetyltransferase Complex to Establish Programs of Cell Proliferation of Crown Root Meristem. The Plant Cell 2017;29:1088–104. https://doi.org/10.1105/tpc.16.00908.

[2] Xu L, Zhao H, Ruan W, Deng M, Wang F, Peng J, et al. ABNORMAL INFLORESCENCE MERISTEM1 Functions in Salicylic Acid Biosynthesis to Maintain Proper Reactive Oxygen Species Levels for Root Meristem Activity in Rice. The Plant Cell 2017;29:560–74. https://doi.org/10.1105/tpc.16.00665.

[3] Wang L, Guo M, Li Y, Ruan W, Mo X, Wu Z, et al. LARGE ROOT ANGLE1, encoding OsPIN2, is involved in root system architecture in rice. Journal of Experimental Botany 2018;69:385–97.

[4] Liu H, Jia S, Shen D, Liu J, Li J, Zhao H, et al. Four AUXIN RESPONSE FACTOR genes downregulated by microRNA167 are associated with growth and development in Oryza sativa. Functional Plant Biol 2012;39:736–44. https://doi.org/10.1071/FP12106.

[5] Kitomi Y, Ogawa A, Kitano H, Inukai Y. *CRL4* regulates crown root formation through auxin transport in rice. Plant Root 2008;2:19–28. https://doi.org/10.3117/plantroot.2.19.

[6] Henderson SW, Wege S, Gilliham M. Plant Cation-Chloride Cotransporters (CCC): Evolutionary Origins and Functional Insights. International Journal of Molecular Sciences 2018;19:492. https://doi.org/10.3390/ijms19020492.

[7] Gao S, Fang J, Xu F, Wang W, Sun X, Chu J, et al. CYTOKININ OXIDASE/DEHYDROGENASE4 Integrates Cytokinin and Auxin Signaling to Control Rice Crown Root Formation. Plant Physiology 2014;165:1035–46. https://doi.org/10.1104/pp.114.238584.

[8] Gao J, van Kleeff PJM, Oecking C, Li KW, Erban A, Kopka J, et al. Light modulated activity of root alkaline/neutral invertase involves the interaction with 14-3-3 proteins. The Plant Journal 2014;80:785–96. https://doi.org/10.1111/tpj.12677.

[9] Zhou H-L, He S-J, Cao Y-R, Chen T, Du B-X, Chu C-C, et al. OsGLU1, a putative membrane-bound endo-1, 4-ß-d-glucanase from rice, affects plant internode elongation. Plant Molecular Biology 2006;60:137–51.

[10] Arai-Sanoh Y, Takai T, Yoshinaga S, Nakano H, Kojima M, Sakakibara H, et al. Deep rooting conferred by DEEPER ROOTING 1 enhances rice yield in paddy fields. Scientific Reports 2014;4:1–6.

[11] Ma N, Wang Y, Qiu S, Kang Z, Che S, Wang G, et al. Overexpression of OsEXPA8, a root-specific gene, improves rice growth and root system architecture by facilitating cell extension. PLoS One 2013;8.

[12] Huang J, Kim CM, Xuan Y, Liu J, Kim TH, Kim B-K, et al. Formin homology 1 (OsFH1) regulates root-hair elongation in rice (Oryza sativa. Planta 2013;237:1227–39.

[13] Qin C, Cheng L, Zhang H, He M, Shen J, Zhang Y, et al. OsGatB, the Subunit of tRNA-Dependent Amidotransferase, Is Required for Primary Root Development in Rice. Frontiers in Plant Science 2016;7.
[14] Li J, Zhu S, Song X, Shen Y, Chen H, Yu J, et al. A Rice Glutamate Receptor–Like Gene Is Critical for the Division and Survival of Individual Cells in the Root Apical Meristem. The Plant Cell 2006;18:340–9. https://doi.org/10.1105/tpc.105.037713.
[15] Jiang H, Wang S, Dang L, Wang S, Chen H, Wu Y, et al. A Novel Short-Root Gene Encodes a Glucosamine-6-Phosphate Acetyltransferase Required for Maintaining Normal Root Cell Shape in Rice. Plant Physiology 2005;138:232–42. https://doi.org/10.1104/pp.104.058248.
[16] Inahashi H, Shelley IJ, Yamauchi T, Nishiuchi S, Takahashi-Nosaka M, Matsunami M, et al. OsPIN2, which encodes a member of the auxin efflux carrier proteins, is involved in root elongation growth and lateral root formation patterns via the regulation of auxin distribution in rice. Physiologia Plantarum 2018;164:216–25. https://doi.org/10.1111/ppl.12707.
[17] Leyser O. Auxin signaling. Plant Physiology 2018;176:465–79.
[18] Xia J, Yamaji N, Ma JF. An appropriate concentration of arginine is required for normal root growth in rice. Plant Signaling & Behavior 2014;9:e28717. https://doi.org/10.4161/psb.28717.
[19] Mao C, Wang S, Jia Q, Wu P. OsEIL1, a rice homolog of the Arabidopsis EIN3 regulates the ethylene response as a positive component. Plant Molecular Biology 2006;61:141.
[20] Munir MZ, Ud Din S, Imran M, Zhang Z, Pervaiz T, Han C, et al. Transcriptomic and Anatomic Profiling Reveal Etiolation Promotes Adventitious Rooting by Exogenous Application of 1-Naphthalene Acetic Acid in Robinia pseudoacacia L. Forests 2021;12:789. https://doi.org/10.3390/f12060789.
[21] Takatsuka H, Umeda M. Hormonal control of cell division and elongation along differentiation trajectories in roots. Journal of Experimental Botany 2014;65:2633–43.
[22] Wang H, Lockwood SK, Hoeltzel MF, Schiefelbein JW. The ROOT HAIR DEFECTIVE3 gene encodes an evolutionarily conserved protein with GTP-binding motifs and is required for regulated cell enlargement in Arabidopsis. Genes & Development 1997;11:799–811.
[23] Chen J, Stefano G, Brandizzi F, Zheng H. Arabidopsis RHD3 mediates the generation of the tubular ER network and is required for Golgi distribution and motility in plant cells. Journal of Cell Science 2011;124:2241–52.
[24] Zheng H, Chen J. Emerging aspects of ER organization in root hair tip growth: lessons from RHD3 and Atlastin. Plant Signaling & Behavior 2011;6:1710–3.
[25] Kieber JJ, Schaller GE. Cytokinin signaling in plant development. Development 2018;145:149344.
[26] Du L, Jiao F, Chu J, Jin G, Chen M, Wu P. The two-component signal system in rice (Oryza sativa L.): a genome-wide study of cytokinin signal perception and transduction. Genomics 2007;89:697–707.

[27] Uga Y, Sugimoto K, Ogawa S, Rane J, Ishitani M, Hara N, et al. Control of root system architecture by DEEPER ROOTING 1 increases rice yield under drought conditions. Nature Genetics 2013;45:1097.
[28] Paszkowski U, Kroken S, Roux C, Briggs SP. Rice phosphate transporters include an evolutionarily divergent gene specifically activated in arbuscular mycorrhizal symbiosis. Proceedings of the National Academy of Sciences 2002;99:13324–9.
[29] Spirin V, Mirny LA. Protein complexes and functional modules in molecular networks. Proceedings of the National Academy of Sciences, vol. 100, 2003, p. 12123–8.
[30] Heyl A, Schmülling T. Cytokinin signal perception and transduction. Current Opinion in Plant Biology 2003;6:480–8.
[31] Wang T, Li C, Wu Z, Jia Y, Wang H, Sun S, et al. Abscisic Acid Regulates Auxin Homeostasis in Rice Root Tips to Promote Root Hair Elongation. Frontiers in Plant Science 2017;8.
[32] Shao Y, Zhou H-Z, Wu Y, Zhang H, Lin J, Jiang X, et al. OsSPL3, an SBP-Domain Protein, Regulates Crown Root Development in Rice. Plant Cell 2019;31:1257–75. https://doi.org/10.1105/tpc.19.00038.
[33] Wu S, Xie Y, Zhang J, Ren Y, Zhang X, Wang J, et al. VLN2 Regulates Plant Architecture by Affecting Microfilament Dynamics and Polar Auxin Transport in Rice. The Plant Cell 2015;27:2829–45. https://doi.org/10.1105/tpc.15.00581.
[34] Zhao Y, Cheng S, Song Y, Huang Y, Zhou S, Liu X, et al. The Interaction between Rice ERF3 and WOX11 Promotes Crown Root Development by Regulating Gene Expression Involved in Cytokinin Signaling. The Plant Cell 2015;27:2469–83. https://doi.org/10.1105/tpc.15.00227.
