# Supplementary material for: Protein-protein interaction (PPI) network analysis reveals important hub proteins and sub-network modules for root development in rice (Oryza sativa)
Source: J Genet Eng Biotechnol. 2023 May 29;21:69. doi: 10.1186/s43141-023-00515-8 (PMC10225403; doi:10.1186/s43141-023-00515-8)
Supplement: Supplementary file 2 — Additional file 2: Supplementary Fig. 1. PPI network module visualization with 100 predicted proteins and 45 seed proteins. [file 43141_2023_515_MOESM2_ESM.pdf]

Figures

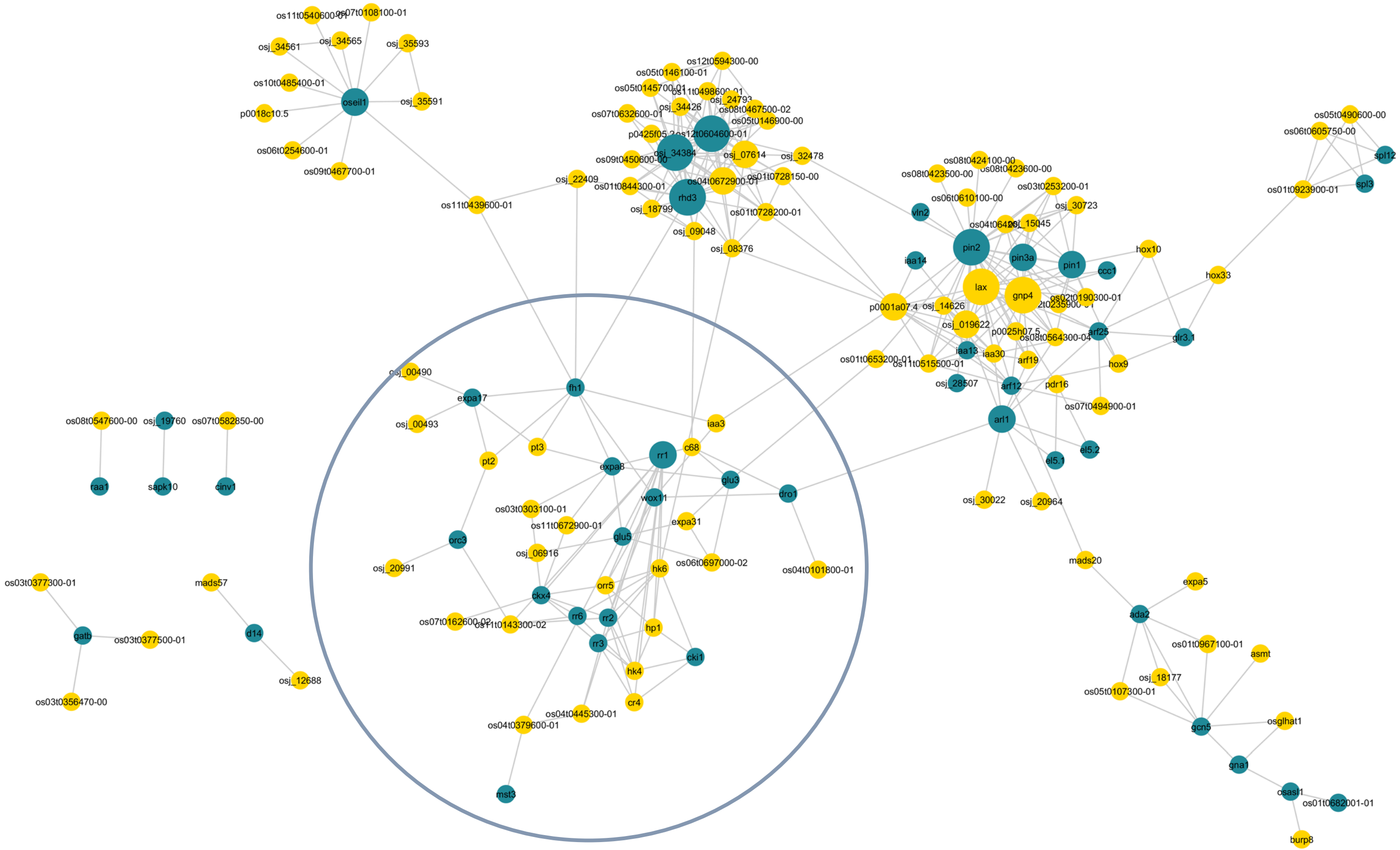

Supplementary Fig. 1. PPI network module visualization with 100 predicted proteins and 45 seed proteins. The Circled cluster indicates the integrated sub-module of sub-modules 3 and 4 in Fig.3. Seeds are represented by the cyan color nodes and predicted candidates are represented by the yellow color nodes.
